# Connecting with nature: The missing link between a satisfied life and a healthy life?

**DOI:** 10.1007/s13280-025-02325-3

**Published:** 2026-01-23

**Authors:** Kate Sollis, Usitha Rajeevan, Lily M van Eeden, Kate Lee, Lucy Keniger, Brenda B Lin, Pauline Marsh, Emily J Flies

**Affiliations:** 1 Geography, Planning and Spatial Sciences, University of Tasmania, Sandy Bay Campus, Hobart, TAS 7001 Australia; 2 Applied Chemistry and Environmental Sciences, RMIT University STEM College, Melbourne, VIC 3000 Australia; 3 Department of Energy, Environment, and Climate Action, Government of Victoria, Arthur Rylah Institute for Environmental Research, Heidelberg, VIC Australia; 4 School of Agriculture, Food and Ecosystem Sciences, University of Melbourne, Parkville, VIC Australia; 5 BehaviourWorks Australia, Monash University, Clayton, VIC Australia; 6 CSIRO, Land and Water Ecosystem Sciences Precinct, 41 Boggo Road, Dutton Park, QLD 4102 Australia; 7 Wicking Dementia Research and Education Centre, University of Tasmania, Sandy Bay Campus, Hobart, TAS 7001 Australia

**Keywords:** Health-related quality-of-life, Life satisfaction, Nature connection, Wellbeing

## Abstract

Nature connection is an important leverage point for human wellbeing and planetary health. In this paper, we examine the varying relationships between nature connection and wellbeing measures through a nationally-representative survey in Australia. We find a positive association between nature connection and two measures of wellbeing: life satisfaction (measured through the personal wellbeing index; PWI) and health-related quality-of-life (measured through the Australian Quality of Life (AQoL) tool). Nature connection was associated with all PWI life domains with a strength of association similar to the relationship between income and PWI. We find a stronger association between nature connection and how *satisfied* a person feels about their life (PWI) than how well a person feels they *function* in life (AQoL). Those with higher levels of nature connection tend to have greater life satisfaction than expected from their health-related quality-of-life. These findings highlight the potential to improve wellbeing via nature connection, by expanding opportunities to meaningfully connect with nature.

**Supplementary Information:**

The online version contains supplementary material available at 10.1007/s13280-025-02325-3.

## Introduction

Nature connection has repeatedly been shown to have positive associations with both wellbeing and pro-environmental behaviours, making it an important leverage point for sustainability (Abson et al. 2017; Richardson et al. 2021; Barragan-Jason et al. 2023). However, global increases in urban living and declines in biodiversity contribute to a growing disconnect between humans and non-human nature, which can be detrimental to human health and wellbeing (Soga and Gaston 2016). Likewise, programs that support human engagement with nature, including caring for or restoring nature, can have multifaceted benefits for both people and environments (Marsh et al. 2023a).

The term’nature connection’ refers to an individual’s relationship with nature—how they think about, feel about, and experience nature or the extent to which humans see themselves as part of the broader natural world (Schultz 2001; Mayer and Frantz 2004; Barragan-Jason et al. 2022; Richardson and Butler 2022). The experiences and expressions of nature connection are as diverse as the definitions of nature itself (Ducarme and Couvet 2020). This is apparent through the various different disciplines through which nature connection has been explored, which include psychology, social sciences, environmental disciplines, tourism, education, planning, and health (Ives et al. 2017). Concepts of nature connection are also closely related to sense of place, which has aspects of emotional attachments, identity, and satisfaction with place for individuals and communities (Manzo 2003; Erfani 2022).

Numerous validated tools have been used to measure nature connection. These include the Inclusion of Nature in Self (INS) scale (Schultz 2001), the Nature Relatedness (NR) scale (Nisbet et al. 2008), the Connectedness to Nature Scale (CNS) (Mayer and Frantz 2004), and the CN-12 (Hatty et al. 2020). These tools use various dimensions to measure nature connection, including cognitive, affective, emotive, philosophical, material, experiential, and behavioural elements (Ives et al. 2017; Hatty et al. 2020; Barragan-Jason et al. 2022; Sollis et al., pre-print).

Direct interactions with nature—spending time in or near nature—have been shown to foster feelings of affinity and connectedness with nature (Schultz and Tabanico 2007), support pro-environmental attitudes and behaviours (Nisbet et al. 2009), and to benefit self-reported wellbeing (White et al. 2019; Martin et al. 2020). Outcome measures reported to be associated with nature connection and nature contact include mental health (Dean et al. 2018; Richardson et al. 2021; Samus et al. 2022; Barragan-Jason et al. 2023), physical health (Richardson et al. 2021; Barragan-Jason et al. 2023), life satisfaction and multidimensional wellbeing (comprising aspects such as material, social, and emotional wellbeing) (Capaldi et al. 2014; Mavoa et al. 2019; Ibáñez-Rueda et al. 2020; Martin et al. 2020). The measures reflecting these outcomes are diverse, ranging from reduced obesity, cardiovascular disease, and mortality to greater overall self-reported health (Keniger et al. 2013; UNEP et al. 2015; Gascon et al. 2016; Twohig-Bennett and Jones 2018; Lai et al. 2019). The evidence for mental health benefits is particularly strong. For example, for people living in built environments, interacting with places with plants or water (‘green’ or ‘blue’ spaces, respectively) has been shown to positively impact on depression, anxiety, stress, and fatigue (Gascon et al. 2016). Additionally, the level of biodiversity in a given area is positively associated with mental health (Buxton et al. 2024).

Like nature connection, wellbeing is increasingly recognised as multidimensional and context-dependent. Wellbeing as a multidimensional construct has been strongly influenced by both the Capability Approach (Sen 1985; Stiglitz et al. 2009) and the Social Indicators movement (Land and Michalos 2018). Both advocate that wellbeing should be measured through a broad set of indicators to reflect the varied and diverse ways that wellbeing can be experienced. These often include material (such as income and housing), relational, psychological, health, and community dimensions (Barrington-Leigh and Escande 2018).

A large body of literature outlines how conceptualisations of wellbeing vary by context (Lu and Gilmour 2004; Tafarodi et al. 2012; Joshanloo 2014; Lomas 2015; Delle Fave et al. 2016; Sollis et al. 2024). For example, research has emphasised how Indigenous Australians have markedly different conceptualisations of wellbeing to non-Indigenous Australians, reflected through the importance placed on relationality, culture, spirituality, and connection to Country (Yap and Yu 2016; Butler et al. 2019). These findings emphasise the importance of ensuring that wellbeing measurement tools adequately reflect the context in which they are being applied.

Furthermore, the way in which wellbeing is measured varies considerably by discipline (Phillips 2006). For example, psychological studies tend to favour hedonic and eudaimonic measures of wellbeing, developed based on ancient Greek philosophies of wellbeing (Ryan and Deci 2001), while the health sciences generally use measures of health-related quality of life (HRQoL) (Karimi and Brazier 2016). This study analyses wellbeing using two types of measures: life satisfaction and HRQoL. Life satisfaction and HRQoL measures are commonly-used instruments to assess the wellbeing of populations. They collectively assess wellbeing from both a subjective standpoint (how satisfied one is with their life), and a more objective, health-focussed standpoint.

Life satisfaction is an evaluative wellbeing measure, firmly rooted in the positive psychology movement which sought to move away from psychology’s traditional focus on mental illness towards wellbeing and flourishing (Pavot and Diener 2008). Life satisfaction measures can be global in nature (asking about life as a whole), or ask about specific domains in life such as health and work (OECD 2013). HRQoL measures typically seek to measure health through a holistic framing, encompassing domains such as physical health, mental health, social relationships, mobility, and the experience of pain (Pequeno et al. 2020). A vast body of literature has identified positive relationships between these two constructs (e.g. Zullig et al. 2005; Siahpush et al. 2008; Kwan 2010; Sun et al. 2016), with this relationship being bi-directional (i.e. life satisfaction enhances HRQoL and HRQoL enhances life satisfaction) (Garrido et al. 2013). Given that these two instruments assess wellbeing from diverse perspectives, this study incorporates both measures.

Despite the growing literature to measure both nature connection and wellbeing through a multidimensional lens, few previous studies have examined the association between nature connection and multidimensional wellbeing. Of those that have, most studies identify strong associations with some (but not all) wellbeing dimensions. For example, Howell et al. (2011) found a significant relationship between nature connection and both psychological and social wellbeing, but not emotional wellbeing. Cervinka et al. (2011) examined wellbeing using the World Health Organization (WHO) Quality of Life Questionnaire (WHOQOL-Bref), finding that nature connection was associated with psychological wellbeing, but not physical wellbeing or evaluation of environmental quality.

In this study, we analyse a national survey with over 4000 participants across Australia to better understand the relationship between nature connection and wellbeing. We contribute to the existing literature base in three important ways. Firstly, we show and compare how nature connection is associated with two measures of multidimensional wellbeing—life satisfaction (measuring through the Personal Wellbeing Index) and health-related quality-of-life (HRQoL, measuring through the AQoL-6D). Secondly, we examine how nature connection moderates the difference between life satisfaction and HRQoL. That is, does nature connection help to explain the difference between life satisfaction (PWI) and HRQoL (AQoL-6D)? Finally, we provide clear policy recommendations on how we can better support individuals to achieve higher wellbeing through enhanced nature connection. This analysis aims to deepen our understanding of opportunities to jointly support humans to connect with nature and enhance the wellbeing of people.

## Materials and methods

### Survey design

An online survey was administered through an existing panel sample run by the Online Research Unit, an online data collection agency in Australia. Individuals are recruited to the panel through both online and offline (post, phone, print) approaches. Members of the online panel were invited to respond to the survey between 6th July and 31st July 2023. The survey comprised of 15 questions related to the individual’s demographics, five questions on human-nature connection (three open-ended questions on experiences of nature, one question administering the Inclusion of Nature in Self scale, and 12 questions administering the CN-12 scale), four questions on nature engagement and environmental behaviours, two questions on wellbeing (the Personal Wellbeing Index, a life satisfaction measure, and the AQoL-6D, a HRQoL measure), and six questions on nature connection and wellbeing across different environments (see Supplementary Information for full survey).

Nature connection was measured using the CN-12 which comprises a series of 12 questions regarding nature connection across three dimensions (identity, experience, and philosophy).^1^ The CN-12 was developed and validated based on a sample of 3090 individuals in the state of Victoria in Australia. The 12 data items were averaged to calculate an overall score as described by Hatty et al. (2020). The CN-12 was identified as the most appropriate primary variable for measuring nature connection due to it being multidimensional and validated with a sample in the Australian context. This is crucial given that conceptualisations of nature connection can vary across cultures (Taylor 2018; Keaulana et al. 2021; Sedawi et al. 2021). For robustness checks, the Inclusion of Nature in Self (INS) developed by Schultz (2002) and a measure of nature contact were also used as predictor variables. These results are presented in Supplementary Information.

Two widely used wellbeing scales, both developed within the Australian context, were used to better understand the relationship between nature connection and wellbeing and compare their explanatory power: the personal wellbeing index (PWI) and the assessment of quality of life-6D (AQoL-6D). The PWI is widely used, both within Australia and internationally, and measures life satisfaction in seven domains of life: standard of living, health, achieving in life, personal relationships, safety, sense of community, and future security (with religion being an optional domain not included in this survey) (International Wellbeing Group 2024). To explore specifically how nature connection is associated with satisfaction regarding quality of one’s local environment, we included an additional data item related specifically to quality of local environment for this survey. We have included this life domain in the analysis of separate PWI life domains, but not in the aggregated index. The aggregated index is a simple average of the individual data items measuring the varied domains.

The AQoL-6D is a health-related quality-of-life measure (HRQoL), developed specifically for the Australian context, and measures six domains of HRQoL: independent living, relationships, mental health, coping, pain, and senses (comprised of elements such as seeing and hearing). An aggregate score for each individual was calculated through a standardised unweighted average of the data items using the methodology provided by Richardson et al. (2012).

This project has been approved by the University of Tasmania Human Research Ethics Committee (project ID 28109). All participants fully consented to being involved in the survey.

### Sample

An initial sample of 4114 individuals took part in the survey, with 108 responses excluded following a data cleaning process. The data cleaning process targeted speeders (respondents who completed the survey in less than 20% of the median completion time), straight-liners (those who gave identical responses across multiple questions), and individuals who provided nonsensical answers to open-ended questions (no respondents were excluded based off this final criterion). Ultimately, 4006 de-identified individuals remained in the final sample, reflecting a 5% response rate typical of large online panels (Daikeler et al. 2020).

The sampling strategy aimed for demographic diversity, including quotas to ensure proportional representation across states and territories (approximately 500 per region), as well as urban and rural areas (50% capital cities, 25% major cities outside capitals, and 25% other regions). State-specific quotas for educational levels were also set to ensure a broad representation, though the quota for the Northern Territory (306 respondents) was not fully met, consistent with its historical survey participation challenges (Australian Bureau of Statistics 2022). Overall, the sample displayed balanced representation across key demographics such as gender, age, education, and income (Table S1 Supplementary Information).

### Analysis approach

#### Examining relationship between nature connection and wellbeing (life satisfaction and HRQoL)

Ordinary least squares (OLS) models were produced to examine the associations between nature connection and two measures of wellbeing (life satisfaction measured through the PWI, and HRQoL measured through the AQoL-6D). Given that both the PWI and AQoL-6D are multidimensional the associations between nature connection and the individual dimensions and domains were also examined (using ordered logit models for the PWI life domains, and OLS models for the AQoL-6D dimensions).

Control variables were based on socio-demographic variables collected in the survey: age, gender, and Indigenous status, whether the individual self-identifies as having a disability, language spoken at home, employment status, highest level of education, personal income, socioeconomic status of area, state of current location, remoteness of current location, and remoteness level respondent grew up in. To test for multicollinearity amongst the independent variables, variance inflation factors (VIFs) were calculated. These ranged from 1.02 to 4.55 which a mean VIF of 1.79 indicating low multicollinearity (Table S2 Supplementary Information).

#### Evaluating the life satisfaction-HRQoL gap

To better understand how the two wellbeing variables (PWI and AQoL-6D) interact and relate to nature connection, a life satisfaction-HRQoL gap was calculated and evaluated. This index was calculated by firstly standardising both the PWI and AQoL-6D to range from 0 to 100. We then calculated the LS-HRQoL gap, which is the difference between the standardised scores of the PWI and the AQoL-6D. OLS models were then produced with the LS-HRQoL gap as the dependent variable with the standardised CN-12 as the independent variable to better understand whether nature connection can help explain some of the variation between the difference in the two outcome variables.

## Results

### Relationship between nature connection and wellbeing

**Fig. 1:**
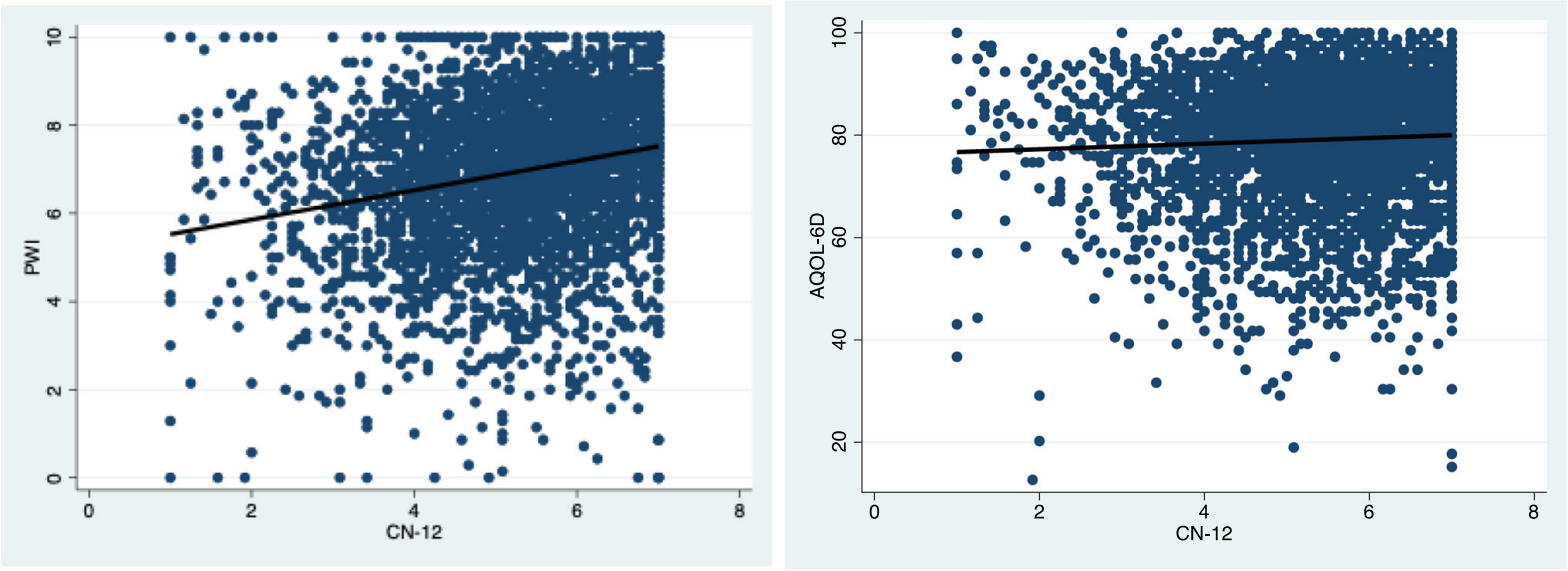
Relationships between CN-12 and wellbeing measures: PWI (left) and AQoL-6D (right)

Scatterplots indicate a linear relationship between the CN-12 with the PWI and AQoL-6D (Fig. 1). Other non-linear relationships were tested, with the model output for a linear relationship showing the best fit. The scatterplots suggest a strong relationship between nature connection and the PWI with a correlation of 0.2310 (*p* < 0.001). While the relationship between the AQoL-6D and CN-12 was significant (*p* < 0.001), it was substantially weaker with a correlation coefficient of 0.0535. The scatterplots also indicate a relatively high level of variation between nature connection and the two measures of wellbeing.

These relationships are further confirmed through OLS models (Table 1) which indicate a significant positive relationship between the CN-12 and the two wellbeing measures when controlling for a range of socio-demographic variables. Similar results were also found when using the INS and nature contact as independent variables (Table S3 Supplementary Information). If we separate the CN-12, PWI, and AQoL scores into quintiles and calculate odds ratios, we find that those in the highest CN-12 quintile are 4.6 times more likely to be in the highest PWI quintile, and 1.9 times more likely to be in the highest AQoL quintile (Table S4 Supplementary Information).

**Table 1:** OLS models identifying predictors of PWI and AQoL-6D. ***Significant at 1% level ** significant at 5% level * significant at 10% level. Standard error in parentheses. Base case is aged over 70, identifies as male, does not identify as Aboriginal and/or Torres Strait Islander, does not have a disability, speaks only English at home, highest level of education is high school completion (Year 12 certificate), employed full time, is in the middle income quintile, currently lives in NSW, lives in a major city, and grew up in a large/capital city

	PWI	AQoL-6D
CN-12	0.325*** (0.0228)	0.638*** (0.163)
Age		
18–30 years	− 0.590*** (0.122)	− 0.857 (0.876)
31–50 years	− 0.781*** (0.116)	− 2.124** (0.828)
51–70 years	− 0.561*** (0.0980)	− 0.455 (0.701)
Gender		
Identifies as female	0.0798 (0.0538)	− 0.773** (0.385)
Non-binary	− 0.310 (0.485)	− 10.24*** (3.467)
Identifies as Aboriginal and/or Torres Strait Islander	− 0.258 (0.184)	0.0142 (1.317)
Has disability/ies	− 1.022*** (0.0944)	− 14.26*** (0.675)
Speaks language other than English at home	0.158* (0.0912)	0.415 (0.653)
Highest level of education		
Has not completed high school (Year 12)	0.128 (0.115)	− 0.837 (0.822)
Certificate/Diploma	− 0.0396 (0.0849)	− 1.043* (0.607)
Undergraduate	0.0667 (0.0870)	0.243 (0.622)
Postgraduate	0.178* (0.0931)	1.177* (0.666)
Employment status		
Part-time	0.0812 (0.0882)	− 0.345 (0.631)
Casual	− 0.125 (0.118)	− 0.274 (0.847)
Self-employed	0.227** (0.113)	1.828** (0.807)
Engaged in home duties/volunteer work	0.318** (0.128)	0.239 (0.914)
Retired	0.546*** (0.0989)	0.894 (0.707)
Not working/studying	0.766*** (0.162)	− 4.185*** (1.160)
Student only	− 0.343 (0.212)	− 1.525 (1.520)
Personal income quintile		
Lowest income quintile	− 0.557*** (0.0995)	− 3.092*** (0.712)
2nd-lowest income quintile	− 0.183** (0.0871)	− 0.668 (0.623)
4th-highest income quintile	0.255*** (0.0825)	1.142* (0.590)
Highest income quintile	0.664*** (0.0943)	4.096*** (0.674)
Standardised IRSAD score	0.0364 (0.0337)	− 1.616*** (0.241)
State/territory		
Victoria	0.178* (0.0996)	0.560 (0.713)
Queensland	0.122 (0.1000)	0.359 (0.715)
South Australia	0.134 (0.101)	0.741 (0.724)
Western Australia	0.104 (0.101)	0.921 (0.720)
Tasmania	− 0.0110 (0.108)	0.00244 (0.773)
Australian Capital Territory	0.107 (0.108)	1.123 (0.774)
Northern Territory	− 0.173 (0.128)	− 2.106** (0.914)
Current remoteness level		
Regional	0.258*** (0.0765)	1.721*** (0.547)
Remote	0.187 (0.156)	2.266** (1.115)
Childhood remoteness level		
Small/medium city	− 0.0589 (0.0701)	− 0.0912 (0.502)
Rural/regional	− 0.0683 (0.0662)	− 0.307 (0.473)
A mix	− 0.499** (0.228)	− 3.828** (1.632)
Constant	5.467*** (0.197)	72.73*** (1.411)
N	3510	3510
*R*-squared	0.196	0.207

To put this finding into context, further analysis indicates that nature connection has a similar level of association with life satisfaction as personal income does. In contrast, income is more strongly associated with HRQoL than nature connection is (Table S5 Supplementary Information).

To better understand what might be driving the positive relationship between nature connection and wellbeing, we analysed separately the different life domains incorporated into the PWI and the different dimensions of the AQoL-6D. The CN-12 has a significant positive relationship with all life domains expressed in the PWI when controlling for a range of socio-demographic characteristics (Table S6 Supplementary Information). For the AQoL-6D, a significant positive association was found between CN-12 and independent living, relationships, coping, and senses (Table S7 Supplementary Information). No association was found between the CN-12 and mental health or pain.

### Exploring the life satisfaction-HRQoL gap

**Fig. 2:**
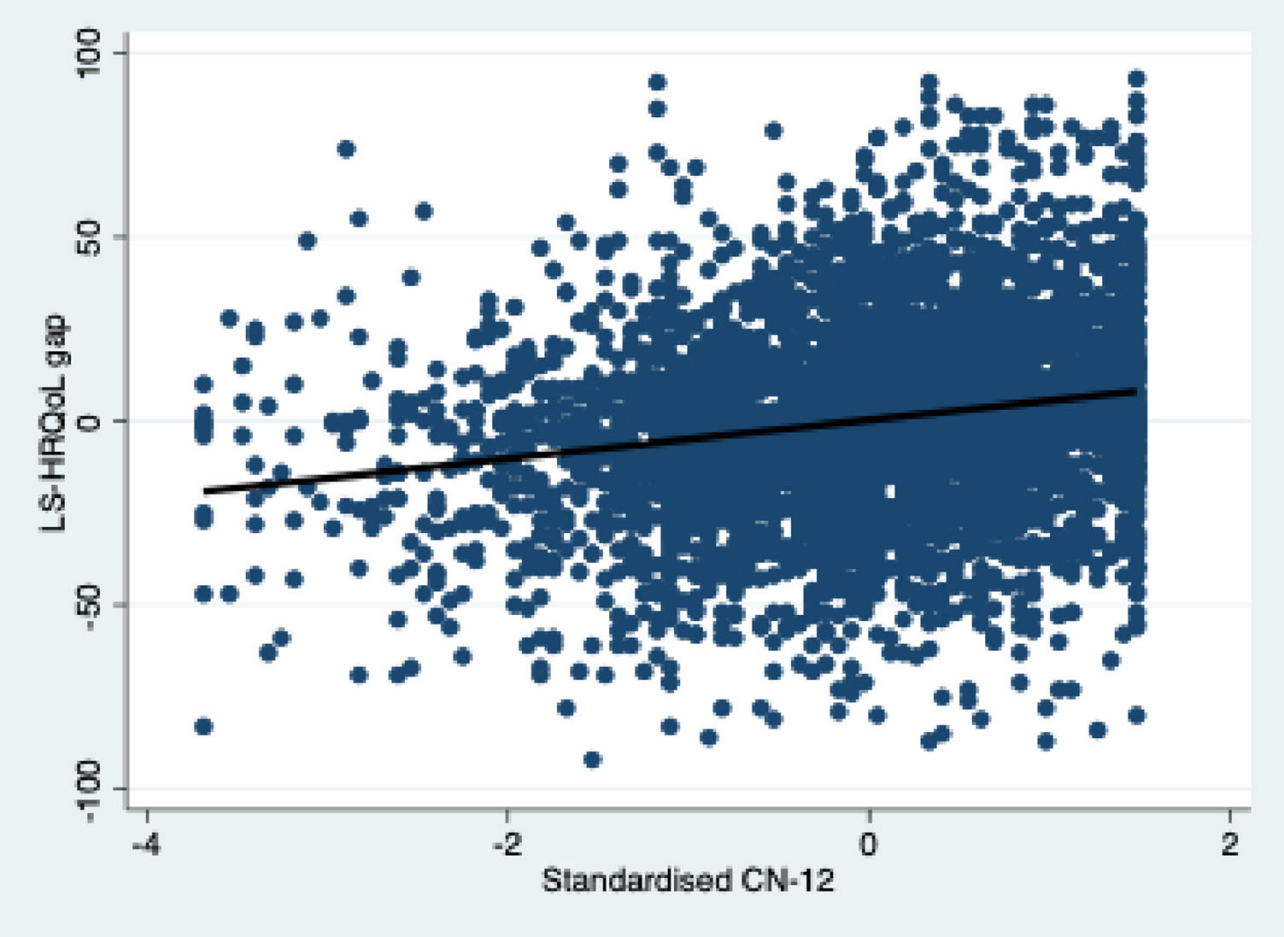
Relationship between the LS-HRQoL gap and the CN-12

To better understand the association between the LS-HRQoL gap and the standardised CN-12, we produced a scatterplot as shown below with a linear line fit to the data (Fig. 2). The scatterplot indicates a positive association. It should also be noted that the mean value (standardised CN-12 = 0) corresponds to a predicted LS-HRQoL gap of just over 0. The interpretation of this is that the mean value of CN-12 predicts no (or a very small) gap between life satisfaction and HRQoL. The further one moves down from the mean value of CN-12, the model predicts a widening LS-HRQoL gap whereby life satisfaction is below HRQoL. The further one moves up from the mean, the model predicts a widening LS-HRQoL gap whereby life satisfaction is greater than HRQoL. In other words, nature connection supports an individual to feel better about their life than their HRQoL status alone would predict.

This is further confirmed through an OLS model (Table S8 Supplementary Information). The model output indicates that the CN-12 has a significant and positive association with the LS-HRQoL gap both on its own and when controlling for several socio-demographic variables. If we separate the CN-12 scores into quintiles and calculate odds ratios, we find that those in the highest CN-12 quintile are 2.2 times more likely to have better life satisfaction than their HRQoL (Table S9 Supplementary Information).

## Limitations

There are important limitations of this study that need to be acknowledged before discussing the results in greater detail. First, it is based on an online panel survey. While all efforts were taken to ensure this survey sample was representative of the diverse groups that make up Australia, it will necessarily exclude people who are less literate with written English and technology. While the sample size was broadly representative of the Australian population, some groups were under-represented. These includes Indigenous Australians, those with high school completion as their highest level of education, and those who speak a language other than English. Though data were screened for fraudulent responses and 108 responses were excluded, there is still the possibility that some included responses were non-genuine.

Second, the measure used to assess nature connection, the CN-12, may inaccurately represent nature connection from non-Western worldviews. The ways of describing nature connection vary across different cultures (Taylor 2018; Keaulana et al. 2021; Sedawi et al. 2021). Thus, the CN-12 may not accurately represent Aboriginal Australian or other Indigenous worldviews of this relationship and likely do not represent other non-Western relationships with nature. A similar critique can be applied to the measures for wellbeing (Christopher 1999; Maulana et al. 2021; Sollis et al. 2024).

## Discussion

The findings presented in this paper strengthen our existing knowledge on the relationship between nature connection and wellbeing. First, we discuss the positive relationship found between nature connection and the two wellbeing measures (PWI and AQoL-6D), particularly in regard to nature connection being associated with every PWI life domain. We then explore nature connection as an important determinant of health.

### The relationship between nature connection and wellbeing

Our findings indicate that nature connection has a significant and positive association with wellbeing when measured through both the PWI and AQoL-6D, even when controlling for a range of demographic factors. This reinforces the findings of previous studies that nature connection may help facilitate higher levels of wellbeing in individuals and across the population. However, the association when applying the PWI is substantially greater than that of the AQoL-6D. This is also a crucial finding, indicating that nature connection has a greater association with evaluative measures of life satisfaction than it does with health-related quality of life in the Australian context.

Previous literature on nature connection and wellbeing finds mixed results based on the measurement tool applied. For example, Martin et al. (2020) found that while nature connection is positively associated with eudaimonic wellbeing (measured through asking about worthwhile activities), it had no association with evaluative wellbeing (a single question on life satisfaction) or general health in a study conducted in England. White et al. (2019) find similar associations using single-item measures of self-reported health and life satisfaction.

To better understand the reason for this divergence, we can firstly look to our analysis by the different life domains within the PWI and the different dimensions of the AQoL-6D (S6 and S7 Supplementary Information). Nature connection was found to be significantly positively associated with every life domain in the PWI, while only with four of the six dimensions in the AQoL-6D (the mental health and pain dimensions showed no significant association). It is important to examine this finding further given the large amount of research, conducted both internationally and in Australia, highlighting the link between nature connection and mental health (Kaplan 2001; Twohig-Bennett and Jones 2018; White et al. 2019; Divya and Naachimuthu 2020; Wicks et al. 2023).

The pathways between nature connection and mental and physical health are complex (Cleary et al. 2017; Dean et al. 2018; Ágoston et al. 2024). Nature connection can be related to a range of eco-emotions that lead to different environmental behaviours (Ágoston et al. 2024). For example, through a study in Brisbane and Sydney in Australia, Chang et al. (2024) found a significant association between nature contact and mental health for those who are highly nature connected and whose engagement with nature was intentional. However, the association was less clear for those who had lower connection with nature even when they visited nature frequently. Additional analysis on our survey data indicates similar findings, with nature contact (measured through how often one spends time in nature) significantly positively associated with the mental health dimension of the AQoL only for those who have higher nature connection (see Table S10, Supplementary Information). Given that much of the literature analysing nature connection and mental health examines nature *contact* rather than nature *connection*, further research should examine the intersecting relationships between nature connection, nature contact, and mental health.

We can also examine the different ways in which the AQoL-6D (a HRQoL measure) and PWI (a subjective, life satisfaction wellbeing measure) are conceptualised to better understand these differences in associations. While HRQoL has historically been difficult to define, broad definitions have included how well a person functions in life, factors that impact an individual’s health, and self-reported wellbeing related to disease or treatment (Karimi and Brazier 2016). In contrast, the PWI reports on subjective evaluations of life satisfaction (International Wellbeing Group 2024). Thus, broadly, the findings from this study indicate that nature connection is more strongly associated with how satisfied a person feels about their life than how well a person feels they *function* in life.

Our finding that nature connection is associated with every PWI life domain is significant. The PWI assesses a person’s self-evaluation across a broad range of areas, including standard of living, health, achieving in life, personal relationships, safety, sense of community, and future security. This is in contrast to the relatively small literature base examining the link between nature connection and multidimensional wellbeing, which has largely found that nature connection is associated with some, but not all, wellbeing dimensions (e.g. Cervinka et al. 2011; Howell et al. 2011). While further research is needed on this, these findings provide an initial indication that nature connection can have wide-reaching impacts on a person’s wellbeing.

### Nature connection as a determinant of health and wellbeing

Our study finds that nature connection can help to buffer satisfaction with life, especially for those who may be facing challenging life circumstances. This association mirrors the inequitable impacts found from social and geographical factors on health and wellbeing outcomes; that is, the conditions of social disadvantage are strongly associated with poor health (Tan et al. 2020). Health geographers have demonstrated the importance of place and space for health, particularly for non-communicable conditions (such as mental ill health, chronic diseases and cancers) (Gatrell and Elliott 2014). Importantly, our findings show that individuals with greater nature connection feel more satisfied with their lives even though they may be experiencing health conditions or symptoms that impact their (health-related) quality of life negatively. Consistent with other studies, for people with lower levels of nature connection, the opposite is true: life satisfaction levels decrease when people are disconnected from nature (Barrable and Booth 2022).

This suggests two exciting possibilities: (1) that nature connection could play an important role in mitigating or moderating the impacts of social and geographical determinants of health and (2) that nature connection is in and of itself a socio-geographical determinant of health. Both possibilities indicate that access to nature needs to be considered an important determinant of subjective wellbeing—alongside the other more established determinants such as education, income, housing, and employment. We do not propose that the provision of nature access replaces the need for adequate social infrastructure to support wellbeing—far from it. However, by ensuring greater nature connection, and giving people the chance to *feel* better, we in turn positively impact other individual and societal (objective and subjective) health and wellbeing outcomes (Diener et al. 2018; Brown and Homan 2023; Saldivia et al. 2023).

## Policy to enhance wellbeing through nature connection

Recognition of the role of nature connection in wellbeing and other facets of life is being increasingly recognised around the world. Indeed, connection with nature has been suggested as a useful indicator for sustainable development globally (Richardson et al. 2022). Global evidence suggests a general decline in connection with nature, although the magnitude of these changes vary across countries (Soga and Gaston 2023). However, the potential influence of strong and coordinated policy has been highlighted in a longitudinal study showing *increasing* nature connection in Singapore (Oh et al. 2020). Strong greening policies are one factor that may help improve the psychological and physical connections one has to nature (Soga and Gaston 2023). Connecting to nature through visiting parks or nature-based activities have been shown to improve social cohesion and a sense of belonging (Keniger et al. 2013; Marsh et al. 2023b). Enhanced nature connection can also result in economic benefits—park visits have been found to generate $US2.1 trillion per year through several factors including reduced healthcare costs (Buckley and Chauvenet 2022). Therefore, policies supporting nature connection could have societal-scale benefits for wellbeing.

Australia’s wellbeing framework *Measuring what Matters* (Australian Government 2023*)* highlights nature connection is a “a critical part of our national identity and an essential part of First Nations culture”. These perspectives sit alongside Australia’s *Strategy for Nature* and its number one goal to “connect all Australians with Nature” (Commonwealth of Australia 2019). While there is much work to do to embed these commitments and priorities into practice, there are some key examples of policy being implemented to support both wellbeing and nature connection. These include social prescribing in health, and eco- or nature-based tourism, being trialled across cities and states in Australia such as Green Adelaide (n.d.) and Local Connections (Victoria State Government 2025).

Recognising the central role of connecting with nature, and reciprocal relationships between people and nature is a deep leverage point for broader and longer-term social transformation (Ives et al. 2018; van Eeden et al. 2023). As noted by Martin et al. (2020, p. 10), “policies that improve accessibility and support people to get out into natural environments are likely to play a key part in achieving health and sustainability objectives”.

There is evidence of such policies in the Australian context, which align across land and water conservation and planning, biodiversity, self-determination of First Nations Peoples, conservation volunteering, environment, and health. For example, *Protecting Victoria’s Environment—Biodiversity 2037,* a 20-year strategy for the future of biodiversity, recognises a central role for people connecting with nature and acting to protect it, while the *Victorian Public Health and Wellbeing Plan 2023–2027* (Victoria State Government 2023) recognises the role of recreation opportunities in nature and the importance of public open spaces. These plans are underpinned by the Victorian Memorandum for Health and Nature (Victoria State Government 2017) advocating for greater collaboration across government, business, and communities for the benefit of future generations, alongside a framework for Healthy Parks Healthy People (Parks Victoria 2020). Supporting policies are also evident at the municipal level (City of Melbourne 2017; Bush and Doyon 2023). These policies provide an exemplar that other governments throughout Australia and internationally can draw on to further enhance nature connection and improve outcomes. Realising their vision relies on consistent meaningful collaboration across research and policy domains of health and nature and in resourcing and implementing actions working with communities to deliver programs on the ground.

## Conclusion

Our study has shown that nature connection has a significant and positive association with two measures of wellbeing: life satisfaction and HRQoL. In particular, nature connection was associated with every measured domain of life satisfaction, indicating the complex and wide-reaching ways in which nature connection and wellbeing are related. Surprisingly, we found that nature connection is not associated with the mental health dimension of HRQoL, which diverges from other research specifically examining the relationships between nature connection, nature interaction, and mental health. This points to the need for greater research around how mental health can be supported across all areas of an individual’s life, of which nature connection may only represent one pathway.

Notably, our findings show that nature connection can help moderate the negative impact that poor health can have on life satisfaction. Taken together, these findings emphasise the potential for nature engagement to support wellbeing and the important role of governments to support opportunities for people to meaningfully connect with nature such as through policies supporting urban greening, nature accessibility, and nature play programs. Doing so will help ensure individuals a lifetime of wellbeing.

## Supplementary Information

Below is the link to the electronic supplementary material.

Supplementary file1 (PDF 1010 KB)

## Footnotes

1 The CN-12 questions asked individuals whether they agreed with the following statements on a 7-point Likert scale: I think of myself as someone who is very concerned about taking care of nature; My relationship to nature is a big part of how I think about myself; I feel uneasy if I am away from nature for too long; I feel right at home when I am in nature; Feeling connected to nature helps me deal with everyday stress; I feel a strong emotional connection to nature; I enjoy spending time in nature; I like to get outdoors whenever I get the chance; Being in nature allows me to do the things I like doing most; Everything in nature is connected (e.g. animals, plants, humans, water, air, land, fire, etc.); Human beings and nature are connected by the same ‘energy’ or ‘life-force’; Human wellbeing depends upon living in harmony with nature.
